# Identification of novel biomarkers based on lipid metabolism-related molecular subtypes for moderately severe and severe acute pancreatitis

**DOI:** 10.1186/s12944-023-01972-3

**Published:** 2024-01-02

**Authors:** Jifeng Liu, Lei Zhong, Yunshu Zhang, Jingyuan Ma, Tong Xie, Xu Chen, Biao Zhang, Dong Shang

**Affiliations:** Department of General Surgery, The First Affiliated Hospital of Dalian Medical University, Dalian, Liaoning China

**Keywords:** Acute pancreatitis, Lipid metabolism, Machine learning, Biomarkers, Molecular clusters

## Abstract

**Background:**

Acute pancreatitis (AP) is an unpredictable and potentially fatal disorder. A derailed or unbalanced immune response may be the root of the disease’s severe course. Disorders of lipid metabolism are highly correlated with the occurrence and severity of AP. We aimed to characterize the contribution and immunological characteristics of lipid metabolism-related genes (LMRGs) in non-mild acute pancreatitis (NMAP) and identify a robust subtype and biomarker for NMAP.

**Methods:**

The expression mode of LMRGs and immune characteristics in NMAP were examined. Then LMRG-derived subtypes were identified using consensus clustering. The weighted gene co-expression network analysis (WGCNA) was utilized to determine hub genes and perform functional enrichment analyses. Multiple machine learning methods were used to build the diagnostic model for NMAP patients. To validate the predictive effectiveness, nomograms, receiver operating characteristic (ROC), calibration, and decision curve analysis (DCA) were used. Using gene set variation analysis (GSVA) and single-cell analysis to study the biological roles of model genes.

**Results:**

Dysregulated LMRGs and immunological responses were identified between NMAP and normal individuals. NMAP individuals were divided into two LMRG-related subtypes with significant differences in biological function. The cluster-specific genes are primarily engaged in the regulation of defense response, T cell activation, and positive regulation of cytokine production. Moreover, we constructed a two-gene prediction model with good performance. The expression of CARD16 and MSGT1 was significantly increased in NMAP samples and positively correlated with neutrophil and mast cell infiltration. GSVA results showed that they are mainly upregulated in the T cell receptor complex, immunoglobulin complex circulating, and some immune-related routes. Single-cell analysis indicated that CARD16 was mainly distributed in mixed immune cells and macrophages, and MGST1 was mainly distributed in exocrine glandular cells.

**Conclusions:**

This study presents a novel approach to categorizing NMAP into different clusters based on LMRGs and developing a reliable two-gene biomarker for NMAP.

## Introduction

Acute pancreatitis (AP) is an inflammatory disease of the pancreas whose course depends on severity. According to the 2012 revision of the Atlanta classification, AP could be classified as mild, moderately severe, or severe [1]. Moderately severe acute pancreatitis (MSAP) is characterized by temporary organ failure, local complications, or an aggravation of a co-morbid condition. Severe acute pancreatitis (SAP) refers to continued organ failure that lasts more than 48 h [1]. Non-mild acute pancreatitis (NMAP), including MSAP and SAP, occurs in 20% of AP patients [2]. Although treatment methods such as early enteral feeding, fluid resuscitation, and organ support therapy are often used in clinical settings, the mortality rate of NMAP can reach 35%, which is much greater than MAP [2]. A derailed or unbalanced immune response may be the root of the disease’s severe course. Stimulation of acinar cells initiates a local immune response, and cells of the innate immune system from the bone marrow, such as monocytes and neutrophils, are recruited to areas of damaged tissue in the pancreas [3]. The immediate response of the immune system to local cellular injury influences the progression of AP. Intervention targeting the immune response mechanisms involved is one of the most promising therapeutic options for AP. Therefore, there is a need to study the immune characteristics in AP and find potentially modifiable risk factors and novel biomarkers for the early identification of high-risk AP individuals.

Hypertriglyceridemia has long been thought of as the third most frequent cause of AP [4]. With more studies being done on dyslipidemia during AP, the connection between hypertriglyceridemia and AP is becoming more and more distinct [4]. Acute renal damage, acute respiratory distress syndrome, and a longer duration of hospital stay are all independently linked to higher blood triglyceride levels during AP, according to a large number of recent clinical investigations [5–7]. The study found that AP patients with hyperlipidemia were at greater risk for more severe pancreatitis [8]. At the same time, it was discovered that strict control of triglyceride concentration after acute pancreatitis presentation decreased the probability of recurrence [8]. In addition, it has been found that hypertriglyceridemia during AP, regardless of etiology, is linked to continuous organ failure, which is a leading decisive factor in AP mortality [9–11]. As a result, it is promising to study the molecular subtypes and develop new diagnostic biomarkers for NMAP patients based on lipid metabolism-related genes (LMRGs).

First, we performed a systematic analysis of the LMRGs and immunological characteristics between normal and NMAP individuals. Then, based on DE-LMRGs, NMAP individuals were split into two lipid metabolism-related subgroups. The differences in immune cell infiltration and key pathways were studied further between the two subgroups. NMAP-specific and cluster-specific genes were identified using weighted gene co-expression network analysis (WGCNA), and the biological roles and pathways of gene enrichment were investigated. A two-gene diagnostic model consisting of CARD16 and MGST1 was discovered by applying the least absolute shrinkage and selection operator (LASSO), random forest (RF), and support vector machine recursive feature elimination (SVM-RFE). The receiver operating characteristic (ROC) curves, nomogram, decision curve analysis (DCA), and calibration curves were used to verify the diagnostic model’s effectiveness. At last, we explored the biological characteristics, single-cell maps, and immunofluorescence of these two model genes and constructed their ceRNA regulatory networks. These findings would improve our comprehension of the lipid metabolism mechanism in AP and offer fresh ideas for the early detection of NMAP.

## Method

### Data preparation

The GSE194331 dataset, including 87 AP patients and 32 normal samples, was obtained from the Gene Expression Omnibus (GEO) database, and the clinical information on the samples can be obtained from previous studies [12]. After excluding the MAP samples, 30 NMAP patients (20 MSAP and 10 SAP) and 32 normal individuals were included in this research. Considering the number of genes and their relevance score, as well as previous studies, we selected 1004 LMRGs (relevance score > 10) from the GeneCards database for this analysis [13, 14]. With adjusted *P* < 0.05 and |logFC|> 2 as the criterion, differentially expressed genes (DEGs) between normal and NMAP samples were found by the “limma” program [15].

### Evaluating the immune cell infiltration

We looked at the differences in the immune microenvironment between people with NMAP and people who are healthy using the CIBERSORT algorithm, which can figure out the percentages of infiltration of 22 different types of immune cells. Individuals with a *p*-value < 0.05 represented that the evaluation of the proportions of 22 immune cell subsets generated by CIBERSORT was accurate. These samples were then utilized for further assessing the variations in immune infiltration between different molecular subtypes [16].

### Cluster analysis

The “ConsensusClusterPlus” package was applied to cluster NAMP samples using the expressed levels of the DE-LMRGs [17]. The reliability of clustering results was demonstrated by PCA. The biological characteristics of different subtypes were further compared.

### Identifying specific genes in key modules

We used the “WGCNA” package in R to find modules and genes that are linked to NAMP and lipid metabolism-related clusters [18]. Using the optimal soft power, the weighted neighbor matrices were built and converted to a topological overlap matrix (TOM). The minimum module size was set to 100, and then the TOM dissimilarity metric was used to construct the modules. Genes with gene significance (GS) > 0.5 and module membership (MM) > 0.8 were considered specific genes [19].

### Functional enrichment analysis

The STRING database was utilized to examine protein interactions using a composite score > 0.15 as a condition to determine the validity of such interactions [20]. Functional enrichment analysis was conducted using the Metascape database [21].

### Identifying the best model genes for NMAP

LASSO, RF, and SVM-RFE were performed to filter genes. LASSO was performed by the R package “glmnet” to avoid overfitting and by 10-fold cross-validation to tune the optimal penalty parameter λ [22, 23]. RF was performed using the R package “Random Forest” (ntree = 500) [24]. The feature importance was determined by the mean decrease Gini index calculated by RF, and genes with relative importance > 1.5 were determined as characteristic genes. Meanwhile, SVM-RFE was employed using the R package “e1071” [25]. SVM-RFE uses the principle of structural risk minimization, which aims to optimize the learning performance by minimizing the empirical error. Then, the hub genes for the following studies were chosen from the intersection of the three subsets. The “pROC” package was used to calculate the ROC curves to evaluate the model’s predictability [26]. The correlation of the model genes CARD16 and MGST1 in peripheral blood was assessed by the GTEx dataset in GEPIA, an online tool for bioinformatics based on TCGA and GTEx [27].

### Establishment of a nomogram

A nomogram integrating model genes was constructed by the “rms” R package [28]. Meanwhile, the calibration and DCA curves were used to evaluate the predictive accuracy of the nomogram [29].

### Analysis of the model genes

Gene set variation analysis (GSVA) was conducted for model genes by the “GSVA” package [30]. The association between model genes and immunochemicals was evaluated based on CIBERSORT results. Meanwhile, using the Human Protein Atlas (HPA: https://www.proteinatlas.org/), immunofluorescence was used to demonstrate cellular localization. The latest version of the HPA database has integrated large datasets with single-cell type level information, and the single-cell type data is integrated into the gene search results page, making it possible for visitors to access all the information from a gene-specific perspective [31]. Therefore, we also examined the single-cell type atlases of the model genes in the pancreatic tissues using the HPA platform.

### Construction of ceRNA network

To discover the ceRNA network that may be regulated by model genes. The TargetScan, miRDB, and miRanda databases were utilized to forecast miRNA-mRNA pairs [32]. Only genes that were simultaneously included in all three databases were considered potential mRNA targets for additional investigation. The spongeScan database was utilized to forecast miRNA-lncRNA pairs [33]. Cytoscape was finally used to visualize the ceRNA network.

## Result

### Dysregulation of LMRGs and immune characteristics in NMAP

**Fig. 1:**
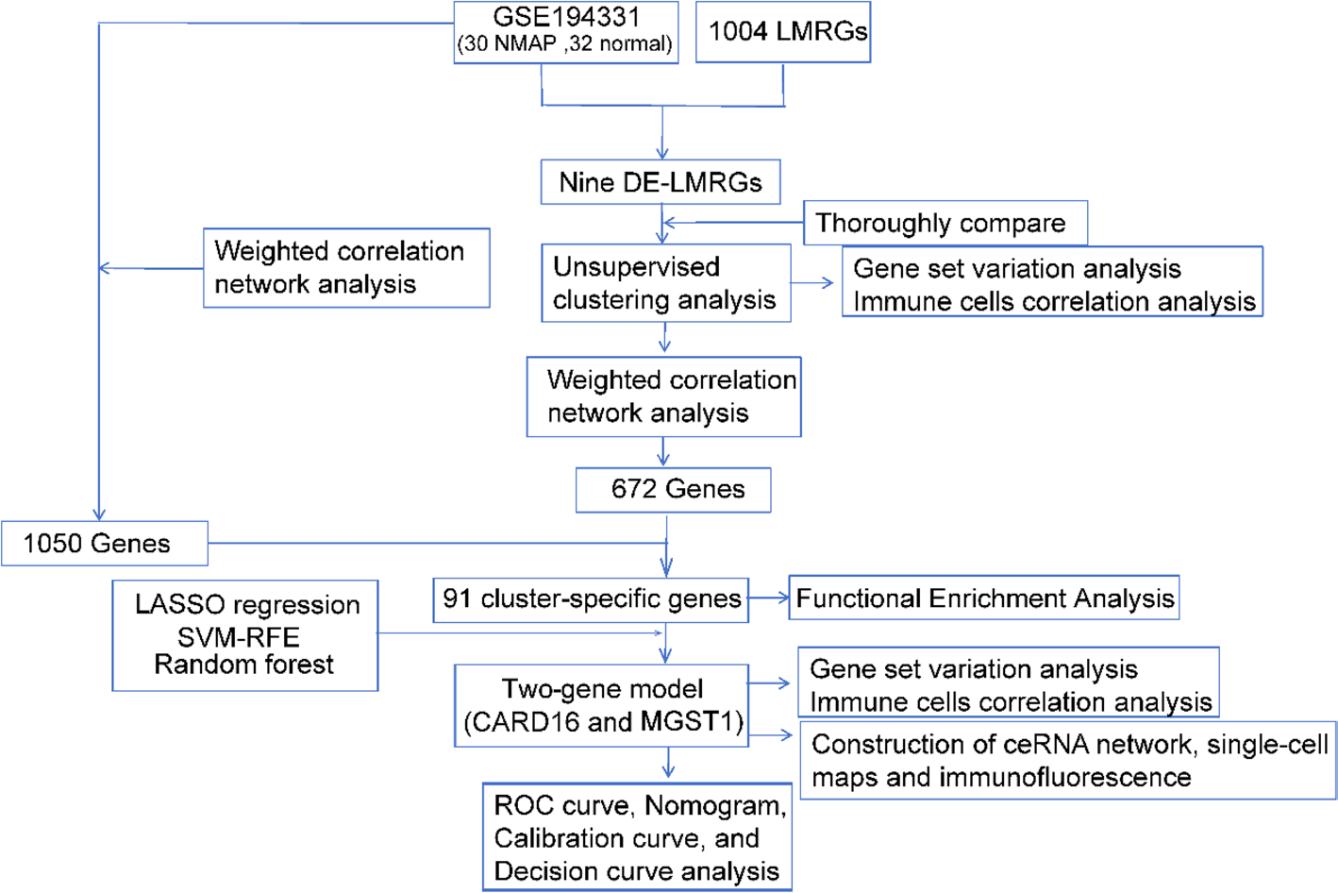
The research’s analytical workflow in detail

**Fig. 2:**
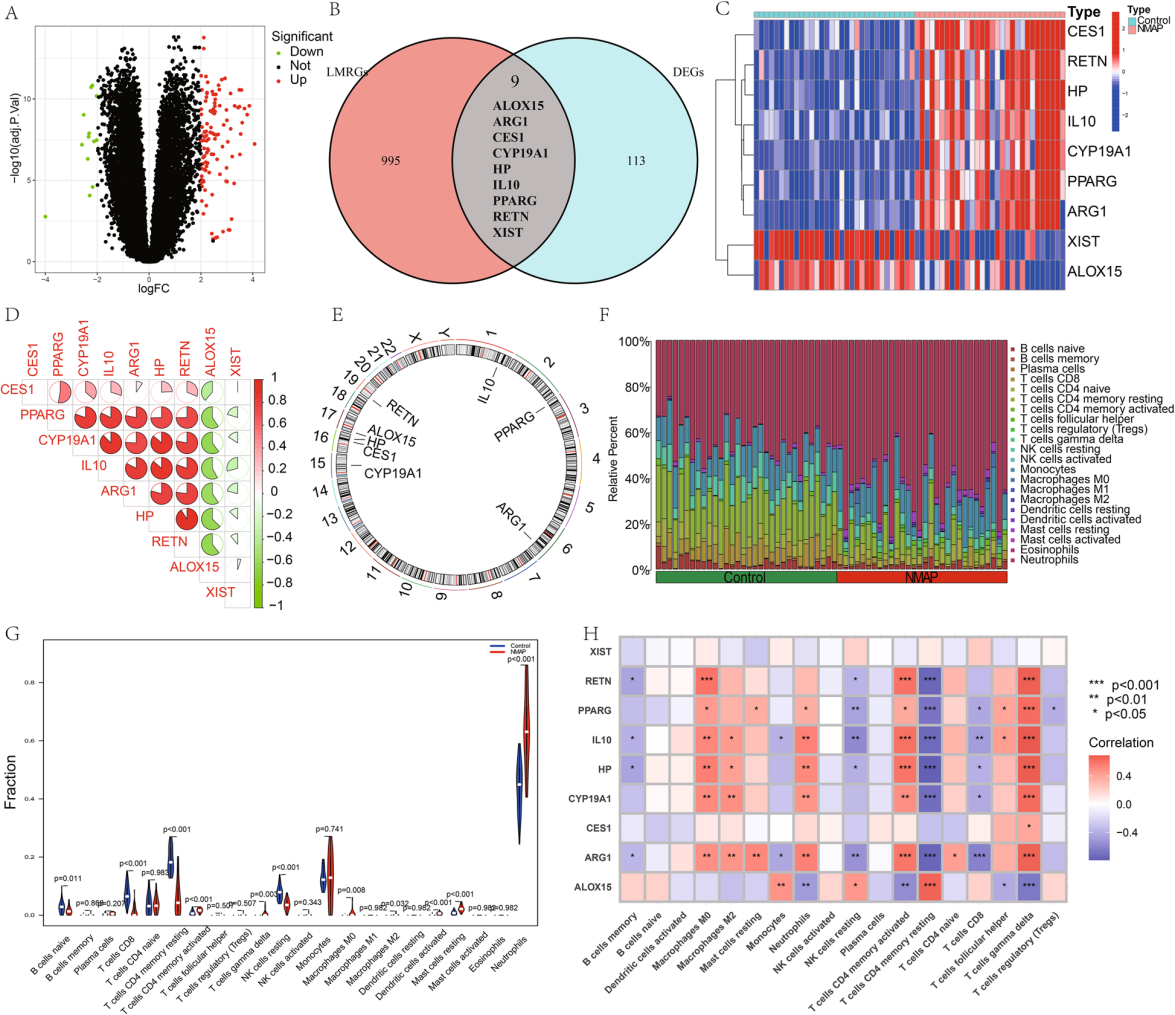
The comparison between NMAP and normal samples. **A** Volcano plot of the DEGs. **B** Venn diagram of the LMRGs and the DEGs. **C** Heatmap showing the expression levels of 9 DE-LMRGs in C1 and C2. **D** The correlation between nine DE-LMRGs. **E** Location of the DE-LMRGs in chromosomes. **F**, **G** Comparison of 22 immune cell infiltration levels between NMAP and normal samples. **H** The relationship between nine DE-LMRGs and immune cells

The current study was illustrated in the flow chart (Fig. 1). Figure 2A illustrates the DEGs between NMAP and normal individuals. We took the intersection of DEGs and LMRGs, and nine LMRGs were found to have differential expression (Fig. 2B). Among them, the expression levels of XIST and ALOX15 were lower, whereas CES1, RETN, HP, IL10, CYP19A1, PPARG, and ARG1 gene expression levels were higher in NMAP than in normal samples (Fig. 2C). Correlation analysis showed mostly positive correlations between DE-LMRGs, except for ALOX15 and XIST (Fig. 2D). Figure 2E showes the location of the DE-LMRGs in chromosomes.

We employed the CIBERSORT algorithm to assess the infiltration proportions of various immune cell subpopulations within each sample and to explore differences in immune infiltration between NMAP and normal samples. It was found that NMAP patients presented higher infiltration levels of T cells CD4 memory activated, neutrophils, and mast cells resting. The number of T cells CD8, T cells CD4 memory resting, B cells naive, and NK cells resting was higher in normal individuals (Fig. 2F and G). Correlation analyses showed that except for CES1 and XIST, the remaining seven DE-LMRGs were significantly positively or negatively correlated with T cells gamma delta and T cells CD4 memory resting (Fig. 2H).

### Identification of LMRG-related clusters in NMAP

**Fig. 3:**
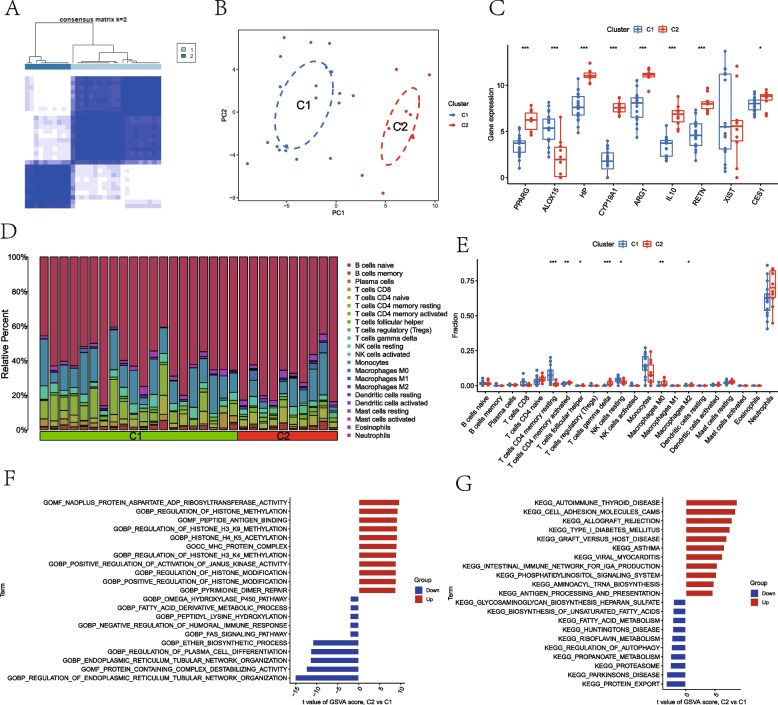
Two LMRG-related clusters in NMAP. **A** NMAP patients are divided into two subgroups. **B** PCA can clearly distinguish two subgroups. **C** The boxplot demonstrates the difference in expression of nine DE-LMRGs between the two clusters. **D**, **E** Comparison of 22 immune cell infiltration levels between the two clusters. **F** Differences in function enrichment between C1 and C2 by GSVA. **G** Differences in the KEGG pathway between C1 and C2 using GSVA. *P < 0.05; **P < 0.01; ***P < 0.001

To further understand the LMRG-related expression patterns in NMAP, we classified the NMAP data based on nine DE-LMRG expression profiles using a consensus clustering technique. When the NMAP individuals were split into two clusters, the results were the most stable (Fig. 3A). A significant difference across the two groups was identified by PCA (Fig. 3B). We looked at the gene expression variations of 9 LMRGs between C1 and C2 to investigate the molecular disparities among clusters. PPARG, HP, CYP19A1, ARG1, IL10, RETN, and CES1 were all strongly elevated in the C2 cluster, whereas ALOX15 was substantially decreased (Fig. 3C). The differences in immune cell infiltration between the two clusters were then compared. We found that the C1 group had a significantly higher abundance of T cells CD4 memory resting and NK cells resting, whereas the C2 group had a significantly higher abundance of T cells CD4 memory activated, T cells gamma delta, and macrophages (Fig. 3D and E). Additionally, we used GSVA to look into possible distinctions in biological function among these two clusters. The findings showed that C1 was primarily involved in the regulation of endoplasmic reticulum tubular network organization, regulation of plasma cell differentiation, and protein export, while C2 was primarily enhanced in antigen processing and presentation, regulation of histone modification, and methylation (Fig. 3F and G).

### Gene module screening and co-expression network development

**Fig. 4:**
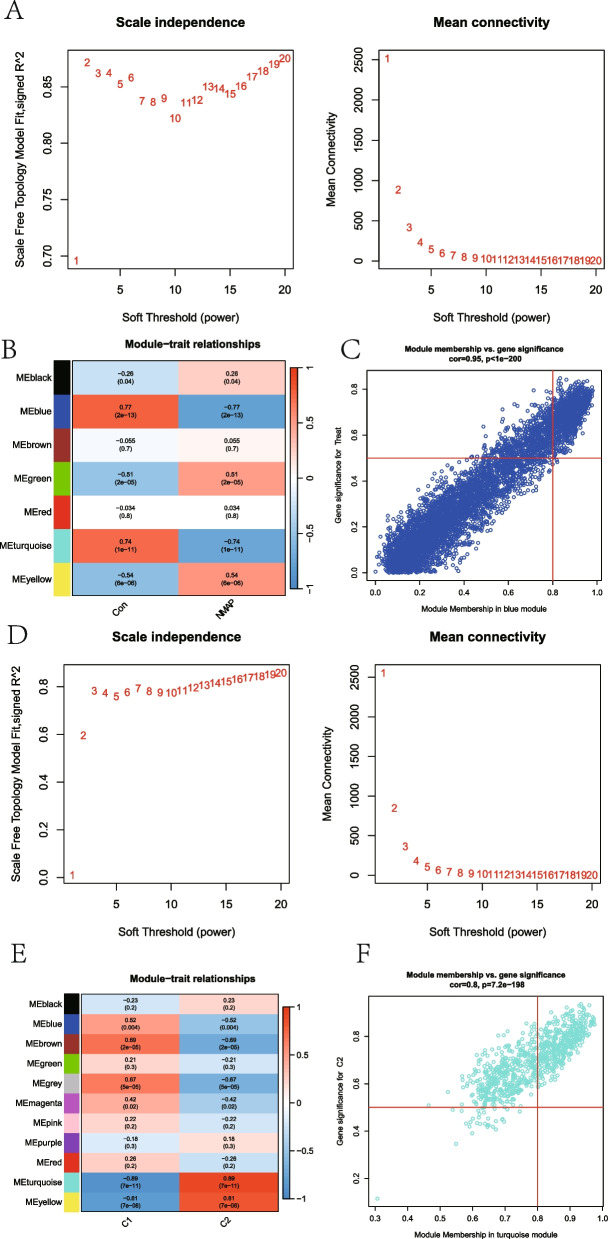
Construction of the co-expressed network. **A** The scale-free fit index and the mean connectivity for various soft-thresholding powers in the first WGCNA. **B** Heatmap showing the correlation between different modules and NMAP traits. The color of the module indicates the correlation between the corresponding module and the trait. **C** Scatter plot of blue module genes. Genes with GS > 0.5 and MM > 0.8 were considered specific genes. **D** The scale-free fit index and the mean connectivity for various soft-thresholding powers in the second WGCNA. **E** Heatmap showing the correlation between different modules and clusters. The color of the module indicates the correlation between the corresponding module and the trait. **F** Scatter plot of turquoise module genes. Genes with GS > 0.5 and MM > 0.8 were considered specific genes

WGCNA was first utilized to discover the important modules related to NMAP. The scale-free R2 parameter was set to 0.9 to identify co-expressed modules (Fig. 4A). The most relevant module was the blue one (Fig. 4B). For further investigation, the hub genes in the blue module were picked (Fig. 4C). Given the important role of lipid metabolism in AP and the significant biological differences between LMRG-related molecule clusters, WGCNA was also used to assess the critical modules that had strong connections to clusters associated with LMRG (Fig. 4D). The LMRG-related clusters were most connected to the turquoise module (Fig. 4E). The crucial genes for the turquoise module were also picked (Fig. 4F).

### Functional enrichment analysis

**Fig. 5:**
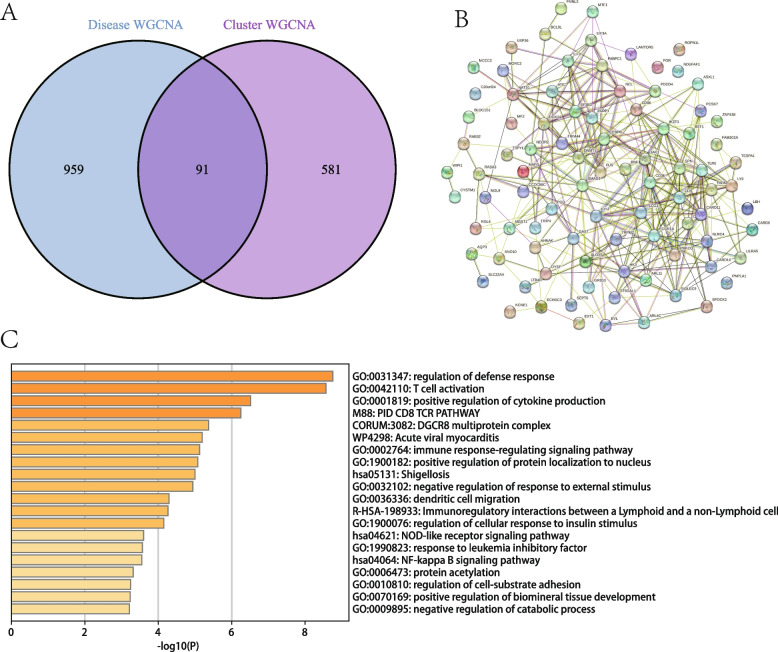
Functional enrichment analysis of hub genes. **A** Taking the intersection of the specific genes from the two cluster analyses yielded 91 hub genes. **B** PPI analysis reveals correlations between hub genes. **C** Functional enrichment analysis of hub genes using the Metascape database

When the module-associated genes of LMRG-related clusters were intersected with the module-associated genes of NMAP, a total of 91 cluster-specific genes were found (Fig. 5A). The PPI analysis showed that most genes were closely linked, except for POR, ROPN1L, NDUFAF1, C20orf24, and PNPLA1 (Fig. 5B). These genes were considerably abundant in the regulation of the defense response and T cell activation, according to the Metascape (Fig. 5C).

### Construction of the model

**Fig. 6:**
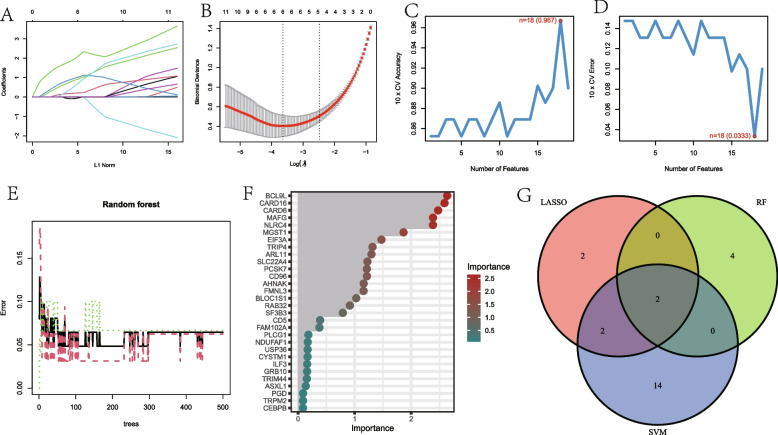
Identifying the best model genes for NMAP by multiple machine learning methods. **A**, **B** The LASSO regression selected 6 genes based on minimum lambda values (**C**, **D**) Identify 18 genes based on SVM-RFE (**E**, **F**) RF ranked the importance of all genes to get 6 genes with scores for importance greater than 1.5. **G** The Venn diagram exhibiting the intersection of three machine learning models

According to the 91 cluster-specific genes, three machine learning models were created. Six genes have been discovered to be possible biomarkers for diagnosis using the LASSO regression technique (Fig. 6A and B). Eighteen genes were extracted from these genes as candidate biomarkers by the SVM-RFE algorithm (Fig. 6C and D). Regarding the RF algorithm, six genes with scores for importance greater than 1.5 were included in the ensuing analysis (Fig. 6E and F). Finally, the two genes (CARD16 and MGST1) were then overlaid by a Venn diagram and used as powerful diagnostic biomarkers (Fig. 6G).

### Evaluation of the model

**Fig. 7:**
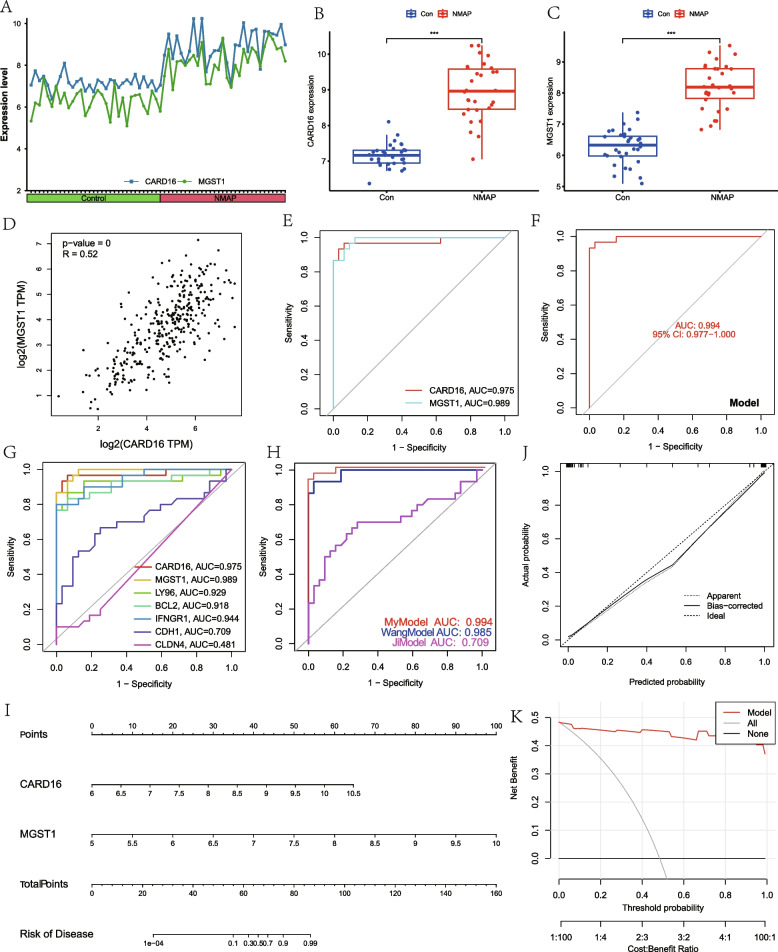
Diagnostic effect of the two-gene model on NMAP. **A** Expression of CARD16 and MSGT1 in different individuals. **B**, **C** Box plots show the expression difference in CARD16 and MSGT1 between NMAP and normal samples. **D** Correlation analysis of CARD16 and MGST1. **E** ROC curves for the evaluation of the CARD16’s and MSGT1’s prediction ability for NMAP. **F** ROC curves for the evaluation of the two-gene model’s prediction abilities for NMAP. **G**, **H** ROC curves of our biomarkers and existing AP diagnostic biomarkers. **I** The nomogram consisting of CARD16 and MSGT1 for forecasting NMAP. **J**, **K** Calibration curve and DCA curve to measure the prediction capability of the model. *P < 0.05; **P < 0.01; ***P < 0.001

Figure 7A illustrates the expression levels of CARD16 and MGST1 in each sample. Boxplots revealed that NMAP samples had substantially greater levels of CARD16 and MGST1 expression than normal samples (Fig. 7B and C). In addition, we analyzed the correlation between CARD16 and MGST1 in normal samples of whole blood using the GTEx dataset in GEPIA. It was found that there was a significant positive correlation between CARD16 and MGST1 (Fig. 7D), suggesting an interaction between the two genes. According to the ROC curves, it can be found that CARD16 and MGST1 both have good predictive value (Fig. 7E). More importantly, the two-gene prediction model demonstrated more favorable performance with an AUC value of 0.994 (Fig. 7F). To further confirm the model’s accuracy, we compared it with the previously published AP diagnostic biomarkers. We found that the AUC of CARD16, MGST1, and our two-gene model was significantly higher than that of existing diagnostic biomarkers (Fig. 7G and H) [34, 35].

In addition, we also created a nomogram to estimate the potential hazards of NMAP individuals (Fig. 7I). The calibration curve demonstrated that there was little variation between the projected and reality risk for NMAP (Fig. 7J). In addition, the DCA curve demonstrated that the overall clinical benefit was greater than in the case in which either all or none of the tests were used for diagnosis (Fig. 7K).

### Biological functions of CARD16 and MGST1 in NMAP

**Fig. 8:**
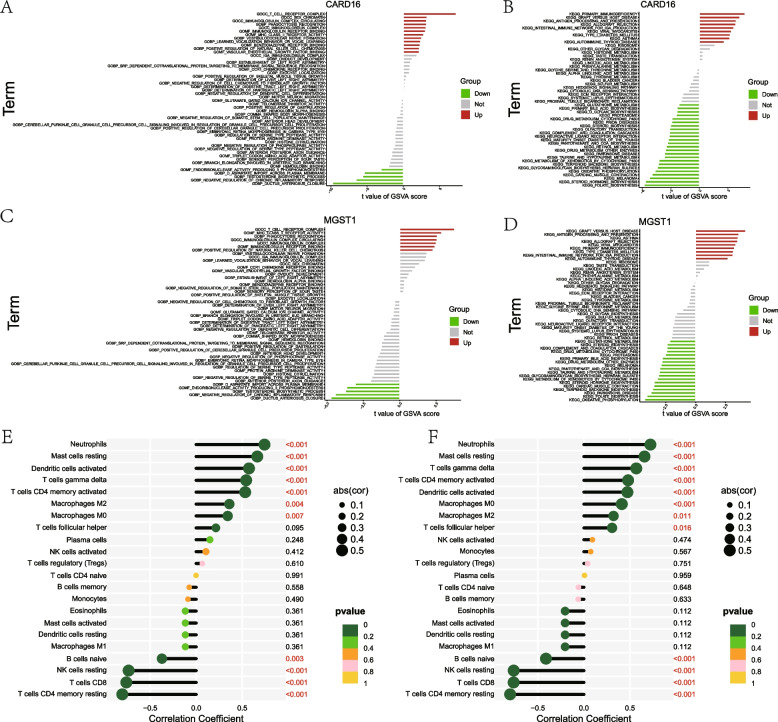
GSVA and correlation analysis of immune cells. Comprehensive scoring of (**A**, **B**) CARD16 and (**C**, **D**) MSGT1 using the GSVA to explore the potential molecular mechanisms by which model genes affect NMAP. **E** Correlations between 22 types of immune cells and CARD16. **F** Correlations between 22 types of immune cells and MSGT1

To investigate the potential functional and molecular mechanisms of CARD16 and MGST1 in NMAP, GSVA was performed. It was found that they both were mainly upregulated in the T cell receptor complex, immunoglobulin complex circulating, as well as some immune-related routes, and downregulated in the negative regulation of chronic inflammatory response and oxidative phosphorylation (Fig. 8A-D). Then, the relationship between two model genes and immunochemicals was further explored. The results revealed that they largely linked negatively with resting NK cells, T cells CD4 memory resting, and T cells CD8 and positively with neutrophils, mast cells resting, and T cells gamma delta (Fig. 8E and F). These results suggest that CARD16 and MGST1 may promote NMAP progression by mediating these inflammatory and immune-related pathways and cells.

### The ceRNA networks, single-cell maps, and immunofluorescence analysis

**Fig. 9:**
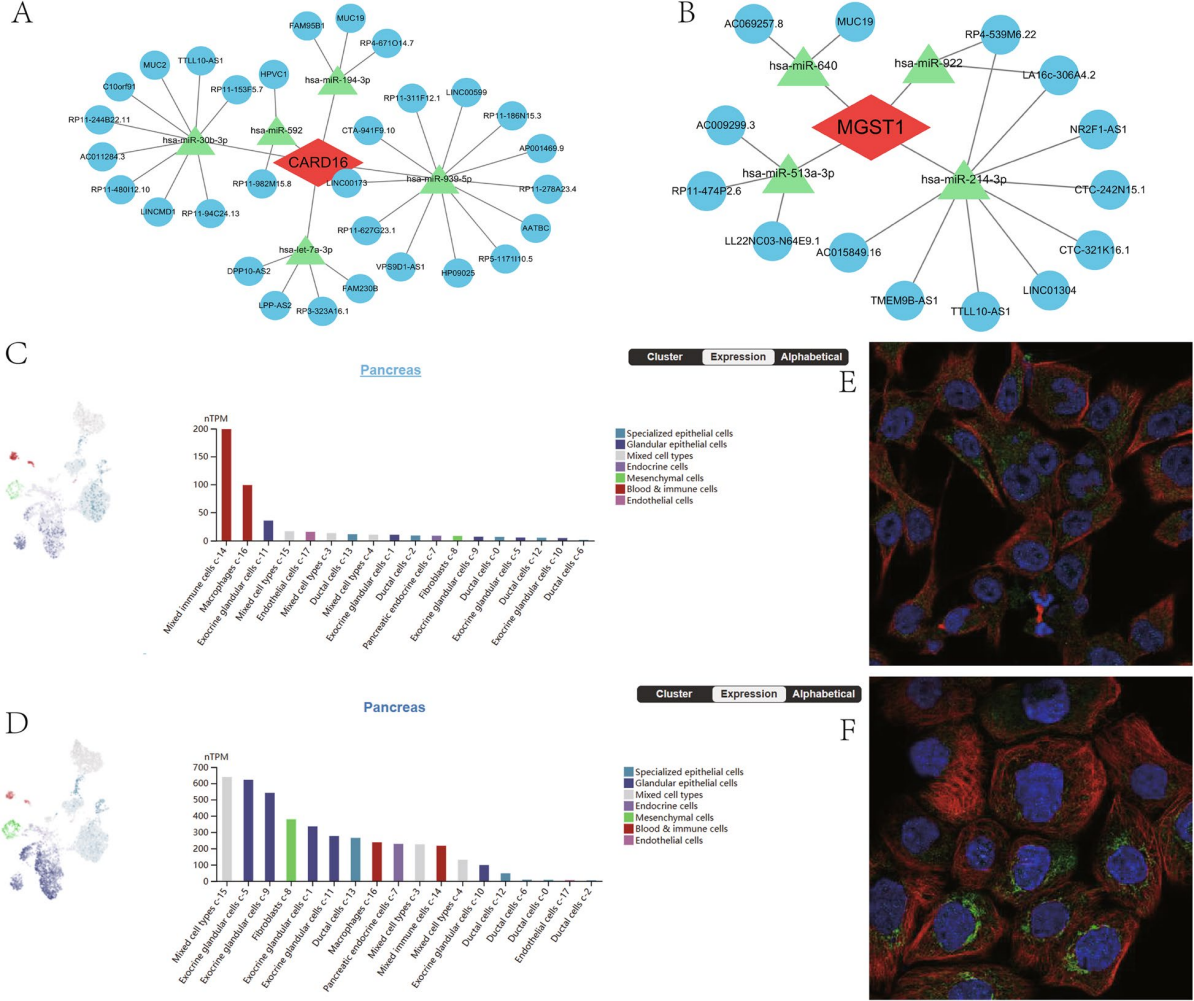
The ceRNA networks, single-cell maps, and immunofluorescence of CARD16 and MSGT1. **A**, **B** The ceRNA networks of CARD16 and MSGT1. **C**, **D** The single-cell type atlases of CARD16 and MSGT1 in the pancreatic tissues. **E**, **F** The immunofluorescence of CARD16 and MSGT1

It is generally accepted that miRNAs bind to mRNAs and then cause gene silence and decrease the expression of genes. However, by binding to miRNA response regions, its upstream molecule, lncRNA, can influence miRNA function and boost the expression of genes. The interactions between RNAs are known as ceRNA networks. Using multiple public databases, CARD16- and MGST1-based ceRNA networks were constructed separately. In the end, we identified 5 objective miRNAs and 30 objective lncRNAs of CARD16 (Fig. 9A) and 4 objective miRNAs and 14 objective lncRNAs of MGST1 (Fig. 9B). The network revealed mechanisms that regulate model genes at the transcriptional level.

Additionally, we analyze the single-cell type for CARD16 and MGST1 using the HPA database. It was found that CARD16 was mainly distributed in mixed immune cells and macrophages, and MGST1 was mainly distributed in exocrine glandular cells (Fig. 9C and D). Then, we explored the cellular localization of CARD16 and MGST1. CARD16 was detected in mitochondria, and MGST1 was detected in mitochondria and endoplasmic reticulum (Fig. 9E and F).

## Discussion

The degree of seriousness of AP, a condition characterized by inflammation, varies greatly, from mild manifestations with a low death rate to serious ones with a high death rate [36]. Therefore, early identification of NMAP is needed to identify individuals who are at risk for organ dysfunction or complications and which patients might benefit from earlier, more intensive treatment. Lipid metabolism has an important impact on AP and is closely related not only to the occurrence of AP but also to its severity. Therefore, it’s valuable to investigate the function of LMRGs in NMAP.

In this research, the gene expression profile of LMRGs in normal and NMAP samples was first thoroughly investigated. There were nine LMRGs identified by differential expression analysis. Immune cells have a major impact on the severity of the AP and are strongly linked to the systemic reaction to pancreas damage [37, 38]. As a consequence, variances in immune cell infiltration among NMAP and normal samples were next contrasted. It was found that NMAP patients had greater infiltration ratios of neutrophils and mast cells. Neutrophils were found to be critical in the development of AP, and treatment aimed at neutrophils significantly reduced tissue damage and prevented pancreatitis [39, 40]. Mast cells also usually play a key role in the inflammatory reaction in AP [41], which is consistent with the results we obtained.

Then, two independent clusters with distinct biological functions were identified to highlight the different patterns of lipid metabolism in NMAP patients. C2 was mainly enriched in antigen processing and presentation, regulation of histone modification and methylation, while C1 was mainly involved in the regulation of endoplasmic reticulum tubular network organization, regulation of plasma cell differentiation, and protein export. Therefore, risk stratification based on LMRG is a potential method for determining prognosis and managing people with NMAP.

Although molecular typing is critical for the functional mining of LMRGs, it is difficult to accurately predict risk scores for specific individuals. To solve this problem, a model consisting of two genes with outstanding performance in predicting NMAP was developed using WGCNA and machine learning techniques. In addition, a nomogram for the diagnosis of NMAP was created using CARD16 and MSGT1. The ROC, DCA, and calibration curves demonstrated the strong predictive value of the model. Meanwhile, we compared it with the previously published AP diagnostic biomarkers. Wang et al. identified three immunogenic cell death-related genes (LY96, BCL2, and IFNGR1) using the same dataset as ours, and Ji et al. identified two genes (CDH1 and CLDN4) that may serve as diagnostic biomarkers for AP using mouse samples. Compared with them, our two-gene model has a significant advantage in the discovery of NMAP.

CARD16, also known as Caspase Recruitment Domain Family Member 16, is a small molecule consisting of 97 amino acids and characterised by a solitary CARD motif. The motif exhibits a sequence identity of 92% with the prodomain of caspase-1 [42]. The CARD16 protein functions as a decoy to hinder the binding of RIP2 to caspase-1 by means of CARD-CARD interaction in the context of the inflammatory response. Consequently, this leads to the suppression of caspase-1 activity and the initiation of NF-κB activation [43]. Furthermore, it has been found that CARD16 plays a role in the stemness maintenance of glioma stem cells and the inhibition of cAMP-induced differentiation [44]. Microsomal glutathione S-transferase 1 (MGST1) is a member of the MAPEG family of membrane proteins. It is a homotrimeric protein with three glutathione binding sites that is found in large amounts in the outer mitochondrial membranes and the endoplasmic reticulum. MGST1 was shown to be an important mediator of inflammation with glutathione S-transferase and peroxidase activities [45]. Emerging data suggest mitochondrial MGST1 may contribute to apoptotic cell death under chemical or oxidative stress [46]. Furthermore, MGST1 exerts inhibitory effects on the process of ferroptosis in cancer cells by interacting with ALOX5, or its lipid peroxidase activities [47]. MGST1 is considered a tumor marker, and its overexpression has been linked to a poor prognosis in several malignancies [48–50], and proposed as a potential therapeutic target for pancreatic cancer [51]. Additionally, it could serve as a biomarker to predict treatment efficacy and assess cancer risk [52, 53]. MGST1 polymorphisms may also influence an individual’s susceptibility to specific cancers [54]. However, no studies have reported the roles of CARD16 and MGST1 in AP, and their potential mechanisms in the development of AP still need to be further explored.

For a deeper investigation of the potential functions and molecular mechanisms of CARD16 and MGST1 of NMAP, GSVA was performed. CARD16 and MGST1 were found to be mainly upregulated in the T cell receptor complex, immunoglobulin complex circulating, as well as some immune-related routes, and downregulated in the negative regulation of chronic inflammatory response and oxidative phosphorylation. Further immune cell correlation analysis identified that the model genes were closely linked to neutrophils and mast cells. Numerous studies have shown a close correlation between neutrophils and the seriousness of AP and that they are crucial to the system-wide inflammatory reaction and other damage to organs brought on by AP. An important factor in determining pancreatic damage and inflammation is increased and persistent neutrophil activation [40]. Mast cells play a significant role in activating local and systemic inflammatory responses in the early stages of the disease [55] and are intimately related to the severity and unfavorable outcomes of AP [41]. These results suggest that CARD16 and MGST1 may promote the activation and recruitment of neutrophils and mast cells through the regulation of the above pathways and functions, thereby promoting pancreatic injury and systemic inflammation.

### Study strengths and limitations

As far as we know, this is the first bioinformatics research to thoroughly examine LMRGs in AP. Given the commonalities of high mortality and concomitant organ dysfunction in MSAP and SAP, both MSAP and SAP samples were included in the present study to enable early diagnosis of NMAP. We innovatively used LMRGs as the basis for grouping and further identified model genes by WGCNA and multiple machine learning methods. However, it is important to recognize that our model validation is still not robust enough, and further basic experimental and clinical studies are still needed. Specifically, blood samples from NMAP and normal samples will be collected for metabolomics analysis to verify the presence of abnormal lipid metabolism in NMAP. The dysregulation of model gene expression in NMAP will be verified by PCR and other methods, and the changes in the levels of AP-related markers will be observed after the knockdown of target genes in AP cell models. Meanwhile, the expression of the target gene in human mast cell lines and monocyte cell lines needed further investigation to clarify its role in immune cells. In addition, the sequencing of clinical samples is needed to validate the predictive ability of our model in a clinical cohort.

## Conclusion

This study presents a novel approach to categorizing NMAP into different clusters based on LMRGs and developing a reliable two-gene diagnostic biomarker for NMAP patients. Importantly, this study may provide a theoretical basis for future studies of AP lipid metabolism.

## Data Availability

The datasets analyzed in this work may be found in the GEO databases. Additionally, any raw data and analytic technologies can be requested by directly contacting the author if the request is reasonable.
